# Acute Intestinal Obstruction—II

**Published:** 1910-09-17

**Authors:** 


### SPECIAL ARTICLE.

### ACUTE INTESTINAL OBSTRUCTION—II.

### Strangulation by Bands.

Passing on now to the next main group of causes of acute intestinal obstruction, the pathological conditions external to the gut and involving it from without may be considered under four headings, namely:—(*a*) Strangulation by bands. (*b*) Strangulation through orifices and internal herniæ. (*c*) Kinking of the gut. (*d*) Tumours pressing on the gut. Of these the first group is by far the most important, for it is one of the commonest causes of acute intestinal obstruction.

(*a*) There are various kinds of bands, *e.g.*:—Peritoneal bands; a Meckel's diverticulum; the appendix vermiformis; a Fallopian tube; appendices epiploicæ; the long pedicle of a tumour, such as an ovarian cyst, or a subserous fibromyoma of the uterus; omental cords; and an empty loop of intestine.

Peritonitis plays a most important rôle in the production of obstruction by bands, for it may be the actual cause of the band, or it may fix the free end of an already existing band or band-like structure, such as the appendix vermiformis, to some other part of the peritoneal cavity in such a manner that a coil of intestine becomes obstructed by it. Considered generally, there are several different ways in which obstruction may be brought about by bands.

1. If a band is short and unyielding, and it is firmly fixed between two points, a loop of intestine may become strangulated beneath it.
2. If it is short and fixed at both ends a coil of intestine may hang over it and become strangulated where it comes into actual contact with the band.
3. If the band is long, whether attached at both ends or free at one end, it may cause obstruction by forming a loop around a coil of intestine.
4. If the band is long and free at one end it may form a knot around a loop of intestine.
5. If one end is attached to a fixed structure such as the brim of the pelvis, and at the other end of a piece of intestine, by contracting it may cause obstruction by pulling on, and kinking the gut.

Peritoneal bands or false ligaments are the result of previous attacks of peritonitis, especially of localised peritonitis such as appendicitis, pelvic peritonitis, tuberculous peritonitis, hernia, after abdominal operations, after injuries, etc. Coils of intestine may at first become adherent to each other or to other structures by means of plastic lymph. After a time normal or increased peristaltic movements lead to stretching of this plastic lymph. Many of the soft, friable cords and bands produced in this way may, of course, give way, but others which are probably thicker and firmer become still more elongated, and, by means of the rolling movements of the intestines, rounded. If organisation occurs there follows a firm, cord-like band, which may stretch between two coils of intestines or between a coil of intestine and some fixed body, such as the brim of the pelvis or the uterus. Such a band may remain fixed at both ends, or one extremity may become so stretched and thinned that it gives way, and a cord attached at one end only results.

The length of these peritoneal bands may vary from about ½ inch to 12 or 15 inches. Treves mentions a case in which a band measured 17½ inches. A case is recorded in which a diverticulum was attached by a band to the mesentery. The band was a thin, fibrous cord, which was attached to the diverticulum an inch from its extremity on one side, and on the other side to the mesentery, close to the origin of the diverticulum. Beneath the fibrous band lay a constricted coil of ileum. The patient was a woman, aged 49, who was admitted for a femoral hernia, with a history of nine days' strangulation. Herniotomy was performed, but the patient died three days after admission, and it was found at the post-mortem examination that the diverticulum of the ileum constricted as above had formed the contents of the hernial sac.

(*b*) *A Meckel's diverticulum* is a blind tube which comes off from the ileum one to three feet above the ileo-cæcal valve; it is due to incomplete obliteration of, or to persistence of the vitelline duct of the embryo. It may vary from a small, almost imperceptible, projection from the ileum to a process nine or ten inches in length. It has the structure of normal intestine. It may cause obstruction in several ways.

If its free end is fixed a loop of intestine may become strangulated beneath it, or hang over it; it may form a loop round a coil of intestine, or even a knot if it is long and its blind end is free and dilated. If its blind end it attached to some fixed structure and there is much traction, it may cause kinking, while if it is short, and it becomes inverted, it may lead to intussusception. The commonest place for a diverticulum to be fixed to is the umbilicus.

### The Appendix Vermiformis and other Structures.

(*c*) *The Appendix Vermiformis.*—The post-mortem examination in a case in which the vermiform appendix was responsible for acute intestinal obstruction showed the appendix to be bent round over the ileum, just above the ileo-cæcal valve, with its apex adherent to the free border of the bowel two feet higher up. At these two points the gut was thus tightly constricted by a band, and the intervening coil of ileum was in the recent state deeply congested. The patient was a man aged forty-six, who was admitted for abdominal pain and vomiting of ten days' duration, which had come on quite suddenly. Death took place three days after admission.

(*d*) *Fallopian tube.*—If the free end of a Fallopian tube becomes attached to some other structure, a loop may be formed under which a coil of intestine may become obstructed. In one such case the right Fallopian tube turned upwards and backwards, and was firmly attached by its fimbriated extremity to the rectum. A loop was thus formed, through which a coil of intestine about six inches in length had passed and become strangulated. Douglas's pouch was obliterated by old adhesions. The patient was a woman aged sixty, who was admitted with intestinal obstruction. The bowels had been confined for three weeks before admission, and for the seven days previous to her coming in vomiting had been well marked. She was operated on, but died on the following day. The strangulated portion of the gut was the ileum, just above the cæcum.

(*e*) *Appendices Epiploicæ.*—The ends of two of the appendices epiploicæ may become adherent, and through the loop thus formed a coil of small intestine may slip and become obstructed, or possibly the appendices may be one on each side of a loop of intestine and become adherent, and contract and produce obstruction in this manner. In one such case the sigmoid flexure of the colon was coated with fat and presented large appendices epiploicæ. Two of these were adherent to each other by their tips, and through the aperture thus formed a loop of ileum five inches in length had slipped and become strangulated.

The patient was a woman aged 48, who was admitted for intestinal obstruction of six days' duration. She was operated upon, but the cause of obstruction was not discovered. She died on the following day. The piece of gut involved was found to be ileum, four inches above the cæcum.

(*f*) *The long pedicle of a tumour.*—Ovarian tumours and uterine fibromyomata often have long pedicles which may act in much the same way as the bands already alluded to, and give rise to acute intestinal obstruction. For instance, a long pedicle may form a loop around a portion of intestine, or, if the tumour becomes fixed, a loop of intestine may become obstructed beneath the pedicle, or may hang over it and thus become obstructed.

(*g*) *Omental cords* may be formed if a portion of the free edge of the great omentum becomes firmly attached to some distant part, such as the brim of the pelvis, the uterus, or a hernial sac or orifice. As a result of this fixation a certain amount of constant traction is exerted on the lower border of the omentum, which consequently becomes stretched, narrowed, and rounded into a band. Bands formed in this way are usually large and thick. Obstruction is produced in the same manner as with other forms of bands.

There is another interesting way in which a band may be produced. A small portion of intestine, as a result of peritonitis, may become attached to a solid or fixed structure in two places close together, and, as a result, a small aperture may be formed, bounded partly by the solid structure and partly by the portion of intestine between its two points of adhesion. A loop of intestine may slip through an aperture thus formed and become strangulated. One remarkable specimen of this kind consisted of a portion of the ileum, the cæcum and appendix vermiformis, and a lithopædion. For 2 feet immediately above the ileo-cæcal valve the ileum was completely collapsed; this was due to strangulation as a result of having passed through a small aperture formed by a short piece of the ileum which had become adherent to the body of the lithopædion in two places. The patient was a woman who was admitted to the hospital for intestinal obstruction. She had been constipated, and had suffered from abdominal pain for ten days previous to her admission. Two and a half years before there was a history of five months' amenorrhœa. As far as she knew, she had never been pregnant. She was now operated upon. A hard mass—the lithopædion—was felt, and considered to be a growth binding down and kinking the intestine.

*Internal Herniæ.*—Strangulation and obstruction may be caused by portions of small intestines slipping through holes or slits in several of the peritoneal structures, *e.g.* in the mesentery, omentum, broad ligament, suspensory ligament of the liver, etc. The causation of these holes and slits is very obscure. Some may be the result of injuries.

Treves mentions a remarkable case in which a loop of the lower part of the ileum was strangulated through a hole in what appeared to be an appendix epiploica. In a case in which a piece of omentum showed a small aperture about an inch in diameter, through which a loop of ileum had slipped and become strangulated, the patient was a man aged 61, who was admitted for symptoms of acute intestinal obstruction of three days' duration. He died three days after admission, and at the post-mortem examination it was found that the last 3 feet of the ileum had become strangulated as a result of having slipped through the aperture in the omentum.

### Internal Herniæ.

In addition to the above, there are the various forms of internal herniæ which may cause acute obstruction. In *diaphragmatic hernia* the opening in the diaphragm may be congenital or it may be due to injury. The organs which are most usually herniated are the stomach, or transverse colon, and, more rarely, the small intestine, liver, or spleen. The physical signs produced are: falling-in of the abdomen, dyspnœa, displacement of the heart, and distension of one side of the chest, with hyperresonance and suppression of the respiratory sounds. The first case of *Hernia into the fossa duodeno-jejunalis*—or, as it is more commonly called, retroperitoneal hernia—was recorded by Treitz in the Guy's Hospital Reports for 1872. The fossa is formed by a fold of peritoneum at the junction of the duodenum with the jejunum. The whole of the small intestine may be implicated. In *Hernia into the foramen of Winslow*, the small intestine slips through the foramen of Winslow into the lesser peritoneal cavity behind the stomach, while in *Intersigmoid Hernia* a funnel-shaped opening, called the intersigmoid fossa, may be formed by the meso-colon over the bifurcation of the left iliac vessels. The symptoms may be acute and very rapidly fatal. Pouches may be formed by the peritoneum in the region of the cæcum. A few cases of hernia into one of these (*perityphilitic herniæ*) are on record.

With reference to the diagnosis of strangulation by bands, the onset is always sudden, and in about 70 per cent. of the cases there is a previous history or evidence of peritonitis. Pain is one of the earliest symptoms, and it is usually severe, and in many of the cases is most intense in the umbilical region. Distension may or may not be present; it depends a good deal on the part strangulated—the higher up the strangulation, the less the distension. Vomiting is another symptom which occurs early, is usually severe, and soon becomes stercoraceous. Collapse and prostration soon appear. Constipation is constant and absolute. Tenesmus is absent.

The various forms of internal herniæ and acute kinking are almost impossible to distinguish from obstruction and strangulation by bands.

*Pressure of tumours.*—A tumour may give rise to acute obstruction by pressure on a portion of the intestine if a sudden enlargement arises in the tumour, *e.g.* from hæmorrhage into its substance, or, in the case of an ovarian with a pedicle, the pedicle becomes twisted and the tumour becomes enormously congested as a result, or, if a tumour suddenly becomes jambed and fixed into the pelvis. This is, however, a very rare cause of acute intestinal obstruction. In the *Medical Review* for October, 1900, an abstract of a paper by Schalita, based on two cases of this nature, is given.

The tumour in the first case appeared to be a hæmatoma connected with the right broad ligament, but it turned out to be a multilocular dermoid cyst, containing a number of blood-clots in addition to fat, bones, hair, and colloid material. The tumour in the second case was an ovarian cyst with a twisted pedicle. The obstruction was relieved by the removal of the tumour.

The treatment in very nearly all cases of acute intestinal obstruction is, of course, relief to the obstruction by surgical means as soon as possible, so that an early diagnosis of "an acute abdominal condition requiring immediate laparotomy" is the main thing to aim at.

